# Prevalence of PER-1- type Extended-Spectrum Beta-Lactamaes in Clinical Strains of *Pseudomonas aeruginosa* Isolated from Tabriz, Iran

**Published:** 2012

**Authors:** Mohammad Taghi Akhi, Yones Khalili, Reza Ghottaslou, Mohammad Aghazadeh, Mohammad hosein i Seroush Bar Hagh, Saber Yousefi

**Affiliations:** 1 Department of Microbiology, Faculty of Medicine, and *Research Center of Infectious Diseases and Tropical Medicine, Tabriz University of Medical Sciences, Tabriz***, ****Iran*; 2 *Department of Clinical Laboratory Sciences, Faculty of Paramedicine, Urmia University of Medical Science*

**Keywords:** Combined Disk Test, Double Disk Synergy Test, Extended- Spectrum Beta- Lactamase, PER-1gene, Pseudomonas aeruginosa

## Abstract

**Objective(s):**

The aim of this study was to investigate the presence of PER-1-type ESBLs in drug resistant *Pseudomonas aeruginosa* isolates.

**Materials and Methods:**

During one-year period (2008-2009), following isolation and identification of 56 *P. aeruginosa, *the *E*-test method was performed for determination of minimal inhibitory concentration of ceftazidim. The isolates that they had MIC≥16 µg/ml against ceftazidim were used for determination of ESBL-producing by combined disk test (CDT) and double disk synergy test (DDST) methods. *Bla*_ PER-1_ gene was investigated by PCR. *P. aeruginosa* KOAS was used as positive control.

**Results:**

Twenty-nine (51.78%) out of fifty six isolates had MIC≥16 µg/ml to ceftazidime, twenty two (75.86%) of them were ESBL producers. Some isolates (27.5%) contained *bla*_ PER-1_ gene.

**Conclusion:**

PER-1-type ESBLs producing *P.aeruginosa* has not been reported previously in but there has been a rather high prevalence of it.

## Introduction

*Pseudomonas* *aeruginosa* is an opportunistic pathogen that causes serious infections among immunity-impaired patients. These bacteria are resistant to many antibiotics intrinsically and are able to produce different virulence factors. Intrinsically resistance of *P. aeroginosa* to different antibiotics is generally due to decreased membrane permeability and efflux pumping (1). Like other Gram- negative bacteria, *P. aeruginosa* can acquire resistance against beta- lactam antibiotics by induction of beta- lactamases. These enzymes are coded either by chromosomes such as class C (Amp C) cephalosporinases and some of class A extended spectrum beta lactamases (ESBLs) or by plasmids. Amongst different classes of beta lactamases, ESBLs are usually able to hydrolyze extended spectrum cephalosporins and monobactams (2). For the first time class A enzymes were reported in Enterobacteriaceae family but since 1990 presence of these enzymes have been frequently found in *P. aeruginosa* (3). PER-1- type ESBLs were the first reported ESBLs ones in *P. aeruginosa* and like most other ESBLs such as TEM and SHV they can hydrolyze different types of beta lactam antibiotics except for carbapenem and cephamycin (4). The PER-1 gene containing isolates were prevalently reported in hospitalized patients from (5). The infection with ESBLs producing *P. aeruginosa* increases morbidity and mortality among hospitalized patients, bringing out many difficulties in treatment of patients and places, plus a significant economic burden on health services. The aim of this study was to investigate presence of PER-1 type ESBLs gene in *P. aeruginosa* isolated from different clinical specimens. 

## Materials and Methods

***Bacterial isolates***

During a year (from July-2008 to July-2009), cultures were made from all clinical samples taken from microbiology laboratory from ICU wards of Imam Reza Hospital in Tabriz, Iran. Samples were taken from patients who sustained trauma and were under invasive procedures such as intubation and tracheostomy. The isolates were identified using conventional bacteriological tests such as positive oxidase and OF (oxidation and fermentation) tests (, ), growth on Cetrimide agar () at 42 ˚C (6). The isolates were then stored at -20^˚^C in trypticase soy broth () containing 20% glycerol. 

***Determination of ceftazidime minimum inhibitory concentration (MIC)***

The MIC of all isolates to ceftazidim was determined by E-test method (AB Biodisk, ) and the results were interpreted according to the Clinical and Laboratory Standards Institute (CLSI) guidelines (7). *P. aeruginosa* ATCC 27853 was used as control for MIC test. 

***Screening for ESBL-producing isolates***

Combined disk test (CDT) (8) and double disk synergy test (DDST) were performed for detection of ESBLs (2). All isolates which showed MIC≥16 µg/ml to ceftazidime were cultured on Muller-Hinton agar (MH) containing cloxacillin (250 µg/ml) (Sigma, USA). DDSTs were performed by placing disks of ceftazidime (CAZ), cefotaxime (CTX), cefepime (CPM), azteronam (ATM) [30 µg each] (MAST Ltd, UK) at a distance of 20 mm (center to center) from a disk containing AMC (amoxicillin/clavulanate 20/10 µg) (MAST Ltd, UK) on MH plates. ESBL production was interpreted when the cephalosporin zone was expanded by the clavulanate. Combined disk tests were also performed by placing disks of ceftazidime, cefotaxime (CTX), cefepime (CPM), CAZ/clavulanic acid, CTX/clavulanic acid and CPM/clavulanic acid [20/10 µg] (MAST Ltd UK) on MH plates. ESBL production was interpreted if the zones produced by the disks with clavulanate were ≥ 5 mm larger than those without inhibitor (8).

***PCR amplification of β-Lactamase genes***

All ceftazidime resistance *P. aeruginosa *isolates were investigated for detection of* bla*_PER-1 _gene by polymerase chain reaction (PCR) method, DNA extraction was carried out by sodium dodecyl sulphate (SDS) protienase K modiﬁed with N,N,N-trimethyl ammonium bromide (CTAB) (9). The DNA amplification program consisted of an initial denaturation step (94 ˚C, 5 min) followed by 35 cycles of denaturation (94 ˚C, 1 min), annealing (52 ˚C, 1 min), and extension (72 ˚C, 1min) steps. A final extension (72 ˚C, 5 min) step was also included in the scheme. PCR was performed in a total volume of 50 µl containing 2.2 mM MgCl_2_, 0.5 µM each of the forward: (5^'^-ATGAATGTCATTATAAAAGCT-3^'^) and the reverse: 

(5′-TTAATTTGG GCTTAGGG-3′) primers (Bioneer, Germany) (10), 0.2 mM dNTPs, 5 µl PCR 10X buffer and 1µl of DNA template, (all the PCR components were purchased from Fermentas Litvania). Eight micro-liter of PCR products were analyzed in 1.1% agarose (Sigma, USA) and the results were observed under UV light (9). *P. aeruginosa* KOAS and *P. aeroginosa* ATCC 27853 (Institute Pasteur, Iran) were used respectively as positive and negative control strains for *bla*_PER-1_ gene.

## Results

**Figure1:**
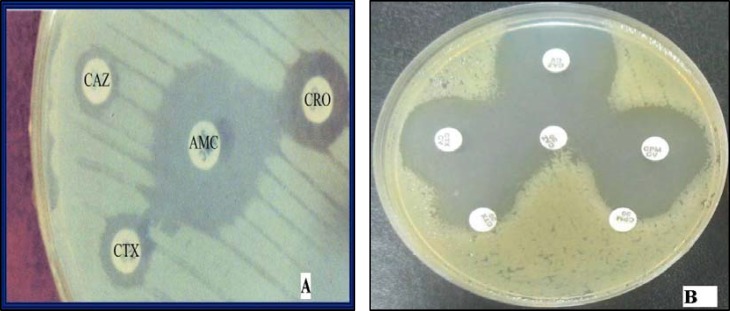
**A-** DDST using ceftazidime (CAZ), ceftriaxone (CRO(, cefotaxime (CTX) and augmentine disks; ESBLs positive *Pseudomonas aeruginosa *showing distinct extension of the zone of inhibition towards AMC. **B-** A representative of *P. aeruginosa* isolates showing a ≥ 5 mm zone size enhancement in the combined disc (CD) test indicating inhibition of ESBL production. A positive CD using different cephalosporins (CAZ, CTX, cefepime CPM) and the same cephalosporins with clavulanic acid (30 μg/10 μg)

**Figure2:**
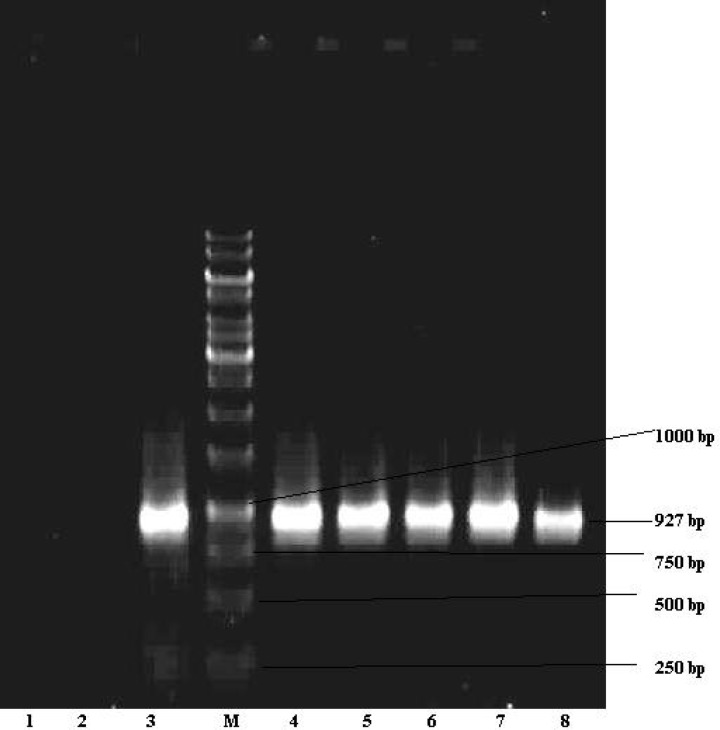
Detection of *PER-1* gene in* Pseudomonas aeruginosa* isolated from clinical specimens (PER-1 specific 927 bp fragment). M; DNA size marker (1 kbp DNA ladder), line 1 negative control *(P. aeruginosa* ATCC 27853), line 2 *PER-1* negative isolate, line 3 Positive control (*P. aeruginosa* KOAS) and lines 4, 5, 6, 7, 8 *PER-1* positive isolates

Fifty six isolates of *P. aeruginosa* were obtained from different clinical specimens such as 23 (41.1%) tracheotomy tube, 17 (30.36%) urine, 7 (12.5%) Bronchoalveolar lavage, 4 (7.14%) blood, 2 (3.57%) sputum, 2 (3.57%) wound and 1 (1.76%) cerebrospinal fluid (CSF). Twenty nine isolates (51.78%) showed MIC≥16µg/ml to ceftazidime. CDT and DDST were carried out on 29 isolates as confirmatory tests for ESBLs production. Twenty two (75.86%) of them showed positive results while 7 (24.14%) isolates were found to not produce ESBLs (Figure-1). It was also found out that eight (27.5%) ceftazidime resistant isolates contained* bla*_PER-1_gene by PCR (Figure 2). 

## Discussion

Extended spectrum cephalosporins are one of the most important antibiotics in treatment of infections produced by *P. aeruginosa* isolates as well as other bacteria in ICU wards (11, 12). Investigations of the ways in which bacteria acquire resistance to these antibiotics are also important and necessary. Phenotypic identification of ESBLs in *P. aeroginosa* by using clavulanic acid for some reasons such as class Amp C cephalosporinase, production of metalo-beta lactamases and oxacillinases (class B & D beta lactamases) and resistant to inhibitory effect of clavulanic acid in some ESBLs type like GES is more difficult than Enterobacteriaceae and often produce false results (3, 13). In order to stop activity of chromosomal Amp C enzymes in ESBLs detection by phenotypic procedure, cloxacillin could be added to the culture media, and it is possible to decrease the distance of disks from 30 mm to 20 mm to increase positive results too (8). Increase of ESBLs prevalence in last decades among *P. aeruginosa* isolates has been proven by several reports from in 2001 (28%) and 2003 (20.6%) (12, 14), in 2005 (25.4%) (11), in 2006 (23.4%) (13), and in 2009 (%40) (10). In this study, we obtained 75.8% ESBLs production by *P. aeruginosa* isolates, which is much higher than the results obtained by others. The reasons for such high results could be the use of improved phenotypic procedure and place of sample collection, as we know in ICU wards antibiotic resistant bacteria are much more prevalent than other wards (12).

Although *bla*_PER-_1 gene possessing isolates are very resistant to beta lactam antibiotics, those which lack this gene, due to having other resistance mechanisms such as efflux pumping and decreased permeability, are also resistant to cephalosporins and monobactams. As far as we know, the study is the first report of PER-1-producing *P. aeruginosa* in Tabriz. For years, PER-1 ß-lactamases were thought to be significant only in Turkey. However, the recent identification of PER-1 producers in several European countries and in the Far East suggests their proceeding dissemination (11, 13, and 14). In Korea, as in Turkey, PER-1 production by *Acinetobacter* spp. has been reported often, but in Europe, it has been identified mostly in *P. aeruginosa*.

The result obtained for prevalence of *bla*_PER-I_ gene in our study is 27.5% (Figure 2), while the results obtained in Turkey have been 11% in 1997 (5) and 55.4% in 2005 (15). Prevalance rate of 13% for* bla*_PER-__I_ gene in Tehran was reported by Mirsalehian *et al* in 2009 (10). PER-I type ESBLs producing *P. aeruginosa* isolates are one of the most important challenges in treatment of infections in Turkey (5, 15).

## Conclusion

Indiscriminate consumption of antibiotics by patients, the high prevalence of *PER-1* gene in Turkey and existence of more communication between Turkey and province of Eastern Azerbaijan (Tabriz) can be the possible reasons for transfer and high prevalence of *PER-*I gene in this region. Of course demonstration of this hypothesis requires typing of these isolates and comparing them with types which are common in Turkey.
